# A Reasonable Limit for Panel Doctors

**Published:** 1913-05-24

**Authors:** J. J. Scanlan

**Affiliations:** Non-panel Representative for the City of London. 1 Castle Court, Cornhill, London, E.C.


*To the Editor of* The Hospital.

### A Reasonable Limit for Panel Doctors.

*To the Editor of* The Hospital.

Sir,—Kindly allow me to make a short reply to your friendly criticism in this week's issue of The Hospital on some remarks of mine, on the above subject, made at the recent meeting of panel and non-panel doctors in the City. The National Insurance Act means that some 14,000,000 of workers in this country have each been given a personal accident and sickness policy, the benefits of which include "free medical attendance." For reasons only too well known, only a certain number of medical men elected to go on the panel, and partly due to this a large number of insured persons are still without a doctor. Other influences are also responsible—viz. rejection or refusal by medical men, who, owing to local conditions, are able to pick and choose their insured members. In some congested industrial centres it is possible for a doctor by selection, according to age, sex, physical condition, and residence, to place several thousand insured persons upon his panel and have comparatively little to do.

Confusion of ideas arises when these thousands of people are described as patients. They are simply insured members, and in some ways may be looked upon in the same light as men in the Army. The Army surgeon, dealing with selected lives, only expects a very small percentage of the men under his care to report themselves on sick parade, and of these the majority are only ill on account of trivial causes. The insurance companies who issue accident and sickness policies (without medical examination) count on a certain percentage of claims only arising, and the basis for the National Insurance Act should be very much the same.

The Commissioners have the power to allocate those insured members who have been refused to be put on panels, and in the same manner it should be possible to adjust matters so that no one doctor should have an excess of insured members belonging to any particular age group if thereby "honest and just treatment" was rendered impossible either in his own or in the practice of a neighbouring colleague. It should be remembered that the "unemployed" contains a large number of individuals who are unemployed because they have been rejected as physically unfit. Medical examination of employees, before and after engagement, by corporations and large companies, is quite the order of the day, and continues to increase in all directions, so that in a large number of cases an insured member must be looked upon as a "selected life."—Yours faithfully,

J. J. Scanlan,
Non-panel Representative for the City of London.

1 Castle Court, Cornhill, London, E.C.,
May 19, 1913.

